# A Review on the Assessment of the Efficacy of Common Treatments in Polycystic Ovarian Syndrome on Prevention of Diabetes Mellitus 

**Published:** 2017-06

**Authors:** Sareh Dashti, Latiffah Abdul Latiff, Nor Afiah Binti Mohd Zulkefli, Anisah Binti Baharom, Halimatus Sakdiah Minhat, Habibah Abdul Hamid, Maimunah Ismail, Ali Jafarzadeh Esfehani, Azrin Shah Abu Bakar, Nur Amirah Inani Binti Sabri

**Affiliations:** 1 Department of Community Health, Faculty of Medicine and Health Sciences, University Putra Malaysia, Serdang, Selangor, Malaysia; 2 Department of Obstetrics and Gynaecology, Faculty of Medicine and Health Sciences, University Putra Malaysia, Serdang, Selangor, Malaysia; 3 Department of Professional Development and Continuing Education, Faculty of Educational Studies, University Putra Malaysia, Serdang, Selangor, Malaysia

**Keywords:** Polycystic Ovarian Syndrome, Diabetes, Metformin, Thiazolidinediones, Lifestyle Modification

## Abstract

**Objective:** Polycystic ovarian syndrome (PCOS) is a common condition amongst women of reproductive age that can result in increased mortality and morbidity in women due to increased risk of diabetes mellitus and cardiovascular diseases. The aim of this systematic review was to assess the effectiveness of common treatments of PCOS on the predictors of diabetes in non-diabetic PCOS women.

**Materials and methods:** An extensive search was performed on the publications in three medical databases including pubmed, scopus and google scholar from 1995 till 2017. The articles were screened based on their quality and included in this systematic review. A total of 25 articles including cohort, randomised controlled trial, review and meta-analysis were included in the review.

**Results:** This systematic review revealed that the effect of lifestyle modification might be low in PCOS subjects due to high drop-out rate while the benefits of this intervention including weight and fat reduction may not be achieved by medical interventions. Metformin treatment may result in improvements in insulin sensitivity while its weight reduction effect is still not documented in PCOS subjects. Thiazolidendiones might be tolerated by the PCOS subjects and may result in similar effects as metformin but this effect should be documented by further studies.

**Conclusion:** Combination of lifestyle modification with metformin or thiazolidinedions might improve the outcome of the prevention strategies. On the other hand this study revealed a different response to treatments in non-obese compared with obese PCOS subjects.

## Introduction

Polycystic ovarian syndrome (PCOS) is defined as hyperandrogenism, anovulation and polycystic ovaries (1, 2). PCOS is a common condition in women at reproductive age (1). The prevalence of PCOS ranges from 3% to 15% in different populations based on the genetic and racial differences as well as the use of different diagnostic criteria and it is estimated that the prevalence would increase by the increasing prevalence of obesity worldwide (1). While PCOS is common in overweight and obese women, it is also seen in about 5% of lean women (3). PCOS is associated with increased risk of type II diabetes and cardiovascular diseases while the burden of PCOS also includes psychosocial problems including depression and anxiety (1).

PCOS is also associated with impaired glucose tolerance, increased insulin resistance and obesity (1, 2). Insulin resistance is believed to be the key factor in the pathogenesis of the PCOS (1, 2, 4, 5). Increased insulin results in the stimulation of ovaries to produce androgens which results in most of the symptoms of PCOS (1, 2, 5). Therefore treatment and prevention of insulin resistance can result in improvement of the symptoms of PCOS as well as preventing the cardiovascular risks of the condition (1, 6). Previous studies on PCOS subjects revealed that BMI, waist circumference, area under curve (AUC) for insulin as well as fasting blood glucose were amongst the predictors of diabetes in this population(7, 8). The aim of this review was to assess the efficacy of the most common treatments of insulin resistance in PCOS on the prevention of diabetes and its related markers in PCOS subjects.

## Materials and methods

A review of the literature was conducted in three medical data bases including pubmed, science direct and google scholar from 1996 till 2017. Data bases were searched in two levels based on defined key words. The keywords for the primary search included poly cystic ovarian syndrome, diabetes, insulin resistance, prevention, lifestyle modification, diet, exercise and treatment. The following MeSH terms were used: Poly Cystic Ovarian Syndrome AND diabetes mellitus OR insulin resistance AND prevention OR control AND life style AND diet AND exercise AND therapeutics. At the second level, database searches were performed based on the findings of the previous level adding more specific keywords including metformin, thiazolidinedione, orlistat and clomiphene to the previous keywords using the following MeSH terms: metformin OR thiazolidinediones AND clomiphene. Articles were then assessed based on title and abstract using the inclusion and exclusion criteria. Inclusion criteria were as follows; all the searched and assessed papers should be in English language (due to the lack of appropriate translator due to insufficient fund), eligible types of studies included randomised controlled trial (RCT), clinical trials, cohort studies, reviews, meta-analyses and cross-sectional studies, the included studies had to perform the following analyses; comparing the effect of treatment options with each other, the case control or RCT studies had to have placebo or control group, studies on larger population with different subjects were included only if they performed sub analysis to compare the response to treatment between treatment modalities. Abstracts and studies with lacking data on the qualitative assessment of searched variables were excluded for the study. Furthermore, studies on pregnant subjects or subjects with the history of smoking, addiction or diabetes were excluded from this systematic review. The screening of the articles took place by two of the authors separately and the selected articles were compared and discussions were performed for the articles that were chosen by one author. Based on the discussion on the eligibility of the study, the study was either included or excluded from the systematic review after the agreement of all authors. Selected articles were assessed for quality based on the STROBE statement checklist (available at http://www.strobe-statement.org). The quality of each article was identified by dividing the total score of each article by 22 (the maximum score of STROBE statement checklist) and was converted to percentage (9). Articles with quality of higher than 70% were included in the review. The data base search resulted in 54 articles among which 30 articles were excluded from the review. A total of 24 articles were included in this systematic review. Due to the observed differences in the results of the studies that were performed on normal weight subjects compared to overweight or obese subjects, the studies were divided into two categories based on the mean body mass index (BMI) of subjects including studies on normal weight subjects and studies on overweight or obese subjects.

**Table 1:** The effect of non-pharmacological interventions on prevention of DM in PCOS subjects

**Author (year)**	**Population**	**Intervention**	**Duration**	**WHR**	**BMI**	**FBS**	**Fasting ** **plasma insulin**	**Insulin ** **sensitivity**
Stamets (2004) (12)	26 Obese PCOS (short term)	13 high protein diet vs 13 high carbohydrate diet	1 month	-	↓	NS	NS	↓
Kasim- Karakas (2009) (14)	24 Obese PCOS (short term)	450 kcal/d diet with simple sugar in 13 subjects vs whey protein in 11 subjects)	2 months	↓	-	-	-	-
Palomba (2008) (11)	40 Obese PCOS (long term)	exercise in 20 subjects vshypocaloric high protein diet in 20 subjects)	24 weeks	↓	↓	-	↓	-

FBS = Fasting blood sugar; g = gram; d = day; Cal = calorie; m = month

## Results

In this review the interventions were divided into two main categories; non-pharmacological interventions including lifestyle modification, pharmacological interventions that include different modalities of medical therapy and combination of non-pharmacological with pharmacological interventions. Moreover, the studies that were performed on non-obese subjects were evaluated separately.

***Non-pharmacological treatments in overweight/ obese subjects***

Lifestyle modification involves either one or a combination of diet or exercise (Table 1, 2) (10-12). Although the type of dietary intervention, study duration, number of subjects and the outcome assessments were different in the studies, preventive effects were found for dietary intervention on diabetes in PCOS subjects (11-14).

**Table 2:** Effect of different diets on diabetes prevention in PCOS subjects

**Type of diet**	**Population**	**Duration**	**WHR**	**BMI**	**FBS**	**Insulin ** **resistance**
High carbohydrate (55% CHO, 15% protein 30% fat)	13	1 month	↓	-	↓	↑
High protein diet (40% CHO, 30% protein, 30% fat)	13	1 month	↓	-	↓	↑
Low calorie diet (450 Kcal) (simple sugar)	16	2 month	-	↓	NS	NS
Low calorie diet (450 Kcal) (whey protein)	17	2 month	-	↓	NS	NS
Standard diet (2000 Kcal) (56% CHO, 16% protein, 31% fat)	11	16 days	-	-	↓	↑
MUFA diet (2000 Kcal) (55% CHO, 15% protein, 30% fat [17% MUFA])	11	16 days	-	-	↓	↑
Low CHO diet (2000 Kcal) (43% CHO, 15% protein, 45% fat [18% MUFA])	11	16 days	-	-	↓	↑
High protein diet (800 Kcal deficit) (45% CHO, 35% protein, 20% fat)	5 with ovulation	6 months	↓	↓	↓	↑
	15 without ovulation	6 months	NS	NS	NS	-

CHO = carbohydrate; MUFA = mono-unsaturated fatty acid; Kcal = Kilocalorie; d = day

In one study, high carbohydrate (55% carbohydrate, 15% protein and 30% fat) and high protein (40% carbohydrate, 30% protein and 30% fat) diets were found to equally reduce weight (-4 kg) in obese PCOS subjects (mean BMI= 37.5 kg/m^2^) (12). The failure to detect any difference in this study was attributed to the small power of the study as well as the small rate of achieving the dietary intake while the quality of carbohydrate and fat content of the diets might have also served as confounders in the study(12). In another study, 2 months administration of a very low calorie diet, 450 kcal/d, with whey protein resulted in a higher BMI reduction compared with very low calorie diet with simple sugar (-1.8 kg/m^2^ in protein group vs -0.5 kg/m^2^ in simple sugar group) in free-living obese PCOS women (BMI=37.2 kg/m^2^) while protein diet was the only diet that resulted in fat mass reduction (-3.1 kg) (14). 

Only one study assessed the effects of diet on insulin resistance on obese PCOS subjects (mean BMI=30.0 kg/m^2^) (13). This study revealed that a 16-day period of low carbohydrate (43% energy carbohydrate) and standard (56% carbohydrate, 31% fat and 16% protein) eucaloric diets (2000 kcal/d) resulted in significant fasting insulin reduction (13). On the other hand low carbohydrate diet and monounsaturated fatty acid (MUFA) enriched diet (17% energy MUFA) resulted in significant acute insulin response to glucose (13).

Only one study compared the effect of exercise and diet in obese PCOS subjects (mean BMI=33.15 kg/m^2^) (11). This study compared the effect of 24 weeks hospital ambulatory based exercise regimen along with maintaining a hypocaloric high protein diet (45% carbohydrate, 35% protein and 20% fat) with each diet and exercise alone (11). The combination intervention was found to reduce BMI, WHR and fasting insulin regardless of the high dropout rates (15% for exercise and 35% for diet intervention) (11). 

***Pharmacological treatments in overweight/obese subjects***

*Metformin: *Although the quantity of the published articles was high (more than 30 articles), a great difference in terms of methodologies was observed in this review. The studies were different in terms of dosage of metformin, duration of the study, sample size, study subjects and especially the BMI of the subjects and presence of other confounders (Table 3).

*Metformin in PCOS: *The main mechanism of action is the inhibition of gluconeogenesis and increasing sensitivity to insulin in liver (16). Metformin can also increase insulin sensitivity by repairing the signaling defects (16, 17). 

The results of the effect of metformin on BMI were controversial. While a RCT on obese PCOS subjects (mean BMI of 34.9 kg/m^2^) found no effect for 1.5 g/d metformin administration for 3 months, larger studies and reviews revealed significant effects for 1.5 to 1.7 g/d metformin administration in reducing area under curve (AUC) for serum insulin after oral glucose administration, BMI and insulin in 1-6 months (18, 19). The difference between the findings of these studies were related to fat mass assessment technique where computed tomography (CT) scan imaging (power of 0.75 for changes in fat mass of 30%) might not have been able to detect small fat mass changes (5-10%) (19). On the other hand, other studies reported that long term (1 to 6 months) administration of 1.5 g/d to 1.7 g/d metformin increased insulin sensitivity (43%) in obese PCOS subjects but did not have any effect on BMI and WHR (Table 3) (15, 20, 21). 

Interesting findings were reported from a RCT on 68 obese or morbid obese (BMI>37 kg/m^2^) PCOS subjects (20). This study revealed that administration of either 1.5 g/d or 2.55 g/d metformin for 8 months resulted in 3.8% reduction in BMI but the weight reduction was more consistent in subjects who received a high dose metformin (20). Furthermore, mild reduction in FBS (-8.3%), fasting insulin (-5.7%) and HOMA-IR (+ 12.6%) was observed only in high dose group (20). The other finding of this study was that the morbid obese subjects significantly responded to both doses of metformin in terms of weight reduction while significant weight reduction was only observed in obese subjects who took higher dose of metformin (20). 

*Thiazolidinediones:* A new class of anti-diabetic agents are Thiazolidinedions (TZD) (22). The main mechanism of action of TDZ is through alterations in gene transcription in adipose tissue, therefore their main effect is through improvement in insulin resistance (22). Although the effect of TZDs was impressive, some of these drugs were withdrawn from the market due to side effects including hepatotoxicity, cardiovascular effects and increased risk of bladder cancer (23-26). 

The effect of thiazolidinediones was found to be different from metformin mainly due to the mechanism of action of these two drugs. In a randomised open label study pioglitazone (45 mg/d) was compared with metformin 1.5 g/d in obese PCOS women (mean BMI=36 kg/m^2^) (27).

**Table 3:** The effect of pharmacological treatments for PCOS on prevention of DM in PCOS subjects

**Body weight ** **category**	**Author (year)**	**Population**	**Intervention**	**Duration**	**WHR**	**BMI**	**FBS**	**Fasting ** **plasma ** **insulin**	**Insulin ** **sensitivity**
Overweight/Obese	Moghetti (2000)*	23 obese PCOS (short term)	Metformin (1.5 g/d) in 14 subjects vs placebo in 9 subjects	6 m	NS	↓	NS	NS	↑
	Nestler (1996)*	11 obese PCOS (short term)	Metformin (1.5 g/d)	4 weeks	NS	NS	NS	NS	↑
	Brettenthaler (2004)	35 PCOS (short term)	Pioglitazone (30 mg/d) in 17 subjectsvs placebo in 18 subjects	3 months	-	NS	NS	↓	↑
	Morin-Papunen (2003)	35 PCOS (short term)	Metformin (n=16)	6 month	↓	↓	-	↓	-
	Harborne (2005)	83 PCOS (long term)	Metformin (1.5 g/d vs 2.55 g/d)	8 months	-	↓	↓	↓	↑
	Jakubowicz (2001)*	48 PCOS (short term)	Metformin (1.5 g/d in 26 subjects vs placebo in 22 subjects)	4 weeks	NS	NS	NS	NS	↑
	Cho (2009)	30 Obese PCOS (short term)	metformin 1.5 g/d vsorlistat 360 mg/d vs pioglitazone 45 mg/d	12 weeks	-	↓In metformin and orlistat groups		↓In orlistat and pioglitazone group	↓In orlistat and pioglitazone group
	Graff (2016)	Meta-analysis on 8 studies (long term)	Metformin vs orlistat	12-24 weeks	-	NS	-	-	NS
Normal weight	Baillargeon (2004)	100 PCOS (short term)	Rosiglitazone (n = 28), metformin (n = 22), combination (n = 20), placebo (n = 30)	6 months	↓	Metformin Rosiglitazone	NS ↓	NS ↑	Combination ↑
	Fleming (2002)*	92 PCOS (short term)	Metformin (1.7 g/d in 47 subjects vs placebo in 45 subjects)	14 weeks	↓	NS	NS	NS	NS
	Kocak (2002)*	46 PCOS (short term)	Metfronin (1.7 g/d in 27 subjects vs placebo in 28 subjects)+clomiphene in both groups	2 cycles	NS	NS	NS	NS	NS
	Yarali (2002)*	32 PCOS (short term)	(metformin 1.7 g/d in 16 subjects vs placebo in 16 subjects)	6 weeks	NS	NS	NS	NS	NS
	Lord (2006)	40 PCOS (short term)	Metformin 1.5 g/d in 21 subjects vs placebo in 19 subjects	3 months	NS	NS	NS	NS	NS

FBS = Fasting blood sugar; g = gram; d = day; Cal = calorie; m = month;

* Studies that were included in the meta-analysis by Lord et al. (2003) (15)

After 12 weeks of follow up, metformin resulted in significant reduction in BMI (-3.4%) compared with pioglitazone (-3.1%), while pioglitazone resulted in significant decrease in HOMA-IR (-41.0%) and insulin level (-37.6%) compared with metformin (-16.1% and -12.8% respectively) (27). In another study, 1.7 g/d metformin resulted in significant reduction in BMI compared with 4 mg/d rosiglitazone (-0.8% for metformin vs 0.2% for rosiglitazone) (28).

A study on PCOS subjects revealed that 4 week administration of 30 mg/d pioglitazone resulted in reduction in fasting insulin level as well as AUC insulin after 3 months of follow up with no significant changes in BMI and WHR (mean BMI of 28.42 kg/m^2^) (29). No serious side effects were reported (29). 

***Other treatments***

Other treatments that are used for PCOS include oral contraceptive, clomiphene and orlistate. 

*Oral contraceptives: *In an open-label controlled trial, 100 overweight PCOS subjects (mean BMI = 36.1 kg/m^2^) were randomised into 4 treatment groups (30). Treatments included metformin 2 g/d (n = 36), high dose oral contraceptive (n = 33) and combination of low dose contraceptive and aldactone (n = 31) (30). The findings revealed that high dose contraceptive resulted in increased insulin resistance (30). Previous reviews also indicated that contraceptives may result in increased insulin resistance in PCOS subjects (6, 31).

*Clomiphene:* In a study on obese women with PCOS by Kocak et al. (2002) clomiphene administration was found to have no effect on BMI or WHR in PCOS subjects (32). As mentioned before clomiphene treatment was shown to increase BMI in PCOS subjects in previous studies (33).

*Orlistat:* Orlistat was also found to be as effective as metformin in a randomised open-label study on 21 obese PCOS subjects (mean BMI = 36.7 kg/m^2^) (28). Another study on obese women with PCOS revealed that 3 months intervention with both 360 mg/d orlistat and 1.5 g/d metformin did not result in changes in fasting insulin or lipid parametrs while orlistat significantly reduced weight (34). Moreover a randomised open label study by Cho et al. (2008) revealed that orlistat (360 mg/d) was the only treatment that could reduce both HOMA-IR in 12 weeks compared with metformin (1.5 g/d) and pioglitazone(45 mg/d) in obese PCOS women (mean BMI = 36.0 kg/m^2^) (27). 

***Combination of pharmacological and non-pharmacological treatments in overweight/obese subjects***

*Metformin with lifestyle modification:* Studies that were performed to assess the effects of combination of metformin and lifestyle modification also reported different findings. These differences were due to the administration of different doses of metformin as well as different lifestyle modification interventions while the sample size and duration of the studies were also heterogeneous (Table 4).

It was shown that PCOS subjects might not be able to maintain their exercise and diet. In a study on morbid obese women with PCOS metformin was found to be more effective than lifestyle modification (either low calorie diet or exercise) (10). In another study on 423 women with PCOS the effect of long term (more than 11 months) administration of metformin was compared with diet (35). The diet was a high-protein (26% of calories), low-carbohydrate (44%) diet (42% of carbohydrate complex) with either 2000 or 1500 kcal/d diet based on the BMI of the subjects and 30% of the calories as fat and a polyunsaturate-saturate ratio of 2:1. Metformin was administered to all subjects at the dose of 2.55 g prescribed as 850 mg 3 times per day with meals. The study revealed that the effect of metformin has a dose dependent effect on prevention of diabetes in PCOS subjects. The authors did not compare the effect of metformin and diet in normal weight women with overweight or obese women (35).

In a study by Glueck et al. (2003) on 138 PCOS subjects amongst which 56 had high fasting blood sugar levels, administration of metformin (2.5 g/d) along with low calorie diet (1500 Calorie diet consisting of 26% protein, 44% carbohydrate and 30% fat) resulted in reduction of WHR but did not result in reduction in the prevalence of DM (based on fasting blood sugar) at 2 and 6 months follow up (36). The complex methodology of the study as well as the presence of PCOS subjects with diabetes reduced the quality of the findings of this study. In a better designed study, 38 obese PCOS women (mean BMI = 36.3 kg/m^2^) subjects were randomly allocated into 4 groups including metformin (1.7 g/d), metformin and lifestyle modification, lifestyle modification and placebo as well as placebo alone for 48 weeks (37). The lifestyle intervention consisted of diet (500-1000 Calorie deficit per day), physical activity (150 minutes per week) (37). The diet included 50% carbohydrate, 25% protein and 25% fat (37). 

**Table 4:** The effect of the combination of lifestyle modification and medications on prevention of DM in PCOS subjects

**Body weight ** **category**	**Author ** **(year)**	**Population**	**Intervention**	**Duration**	**WHR**	**BMI**	**FBS**	**Fasting ** **plasma ** **insulin**	**Insulin ** **sensitivity**
Normal weight	Pasquali (2000)^*^	20 PCOS (long term)	PCOS (metformin 1.7 g/d + low calorie diet 1200- 1400 Calories) vs 20 age and BMI matched non- PCOS subjects	6 months	NS	NS	NS	↓	NS
	Dereli (2005)	40 PCOS (long term)	Rosiglitazone (2 mg/d vs 4 mg/d) + weight maintenance diet	8 months	-		↓	↓	↓
Overweight/Obese	Glueck (2003)	138 PCOS (56 impaired FBS) (short term)	Metformin (2.5 g/d) + diet (1500 Cal/d)	6 m	↓	-	NS	-	-
	Hoeger (2004)	35 PCOS (long term)	Metformin (1.7 g/d in 9 subjects vs lifestyle + placebo in 11 subjects vs lifestyle + metformin in 9 subjects vs placebo in 9 subjects)	48 weeks	-	↓	NS	NS	NS
	Stamets (2004)	36 Obese PCOS (short term)	13 high protein diet vs 13 high carbohydrate diet	1 month	-	↓	NS	NS	↓
	Kasim- Karakas (2008)	24 Obese PCOS (short term)	450 kcal/d diet with simple sugar in 13 subjects vs whey protein in 11 subjects)	2 months	↓	-	-	-	-
	Hoeger (2008)	79 obese PCOS (long term)	metformin 1.7 g/d in 6 subjects, lifestyle modification in 8 subjects, oral contraceptive in 10 subjects and placebo in 10 subjects)	24 weeks	-	↓In oral contraceptive group	↓In metformin group	NS	NS

FBS = Fasting blood sugar; g = gram; d = day ; Cal = calorie; m = month

The study revealed that while the drop-out was high after 24 weeks (34%) no significant difference was found for study variables after 24 weeks (37). 

*Orlistat compared with metformin:* In a systematic review and meta-analysis on 8 studies in 2016, orlistat and metformin were found to equally reduce the body weight of overweight or obese women with PCOS (38). The meta-analysis also revealed no significant difference between the two treatments in weight and BMI reduction (38). The study included only two articles that compared the effects of metformin with orlistat in weight reduction.

*Pharmacological treatments in normal weight subjects:* It is hypothesized that metformin administration has a different effect on obese and non-obese PCOS subjects. A RCT indicated that metformin (1.7 g/d) administration for 6 month reduced WHR, weight, insulin, FBS and HOMA-IR more predominantly in obese (mean BMI = 37.2 kg/m^2^ and n = 40) compared with non-obese (mean BMI = 25.3 kg/m^2^ and n = 16) PCOS subjects (39). In contrast, another study revealed that the morbid obese women (BMI > 37 kg/m^2^) did not respond to metformin administration in terms of WHR and BMI reduction compared with leaner women (BMI < 31 kg/m^2^) (40). There were four other studies that assessed the effect of metformin on women with lower BMI (BMI>28 kg/m^2^) but the results of these studies were not reliable. In one of these studies non-PCOS subjects were taken as control group (41) while the other study was of short duration (6 weeks) (42). The findings of two other studies could not be interpreted due to the use of clomiphene and oral contraceptives (32, 43). It was previously shown that clomiphene treatment could result in increased weight gain compared with combination therapy of metformin and clomiphene in PCOS women (33). The findings of the other study were also of less importance due to the effects of oral contraceptives on insulin resistance and blood sugar (15). None of these studies were strong enough to identify the effect of metformin on weight reduction and fat distribution in PCOS women with lower BMI. 

*Thiazolidinediones:* In another study two doses of rosiglitazone (2 mg/d and 4 mg/d) were assessed in non-obese PCOS subjects (mean BMI of 24.05 ± 1.63 kg/m^2^) (44). Subjects were also asked to follow a weight maintenance diet during the course of study (8 months) in order to neutralise the effect of weight gain in the study (44). Although a dose related response was found for rosiglitazone in OGT, FBS and fasting plasma insulin, it could not significantly reduce BMI, FBS, IGT, plasma insulin and insulin resistance as well as insulin levels after glucose ingestion at different doses (44).

*Thiazolidinediones and metformin combination:* Only two studies were eligible for the systematic review which assessed the effect of thiazolidinedions and metformin combination and both studies were performed on normal weight subjects. A non-significant increase in BMI (0.4%) was occurred in combination therapy which was higher than monotherapy with each drug (28). None of the treatment methods were superior to the other in terms of WHR reduction nor were different from the placebo group in terms of FBS reduction (28).

A RCT on 100 non-overweight PCOS women (mean BMI of 24.6 kg/m^2^), administration of combined metformin (1.7 g/d) and rosiglitazone (4 mg/d) resulted in a significant reduction in WHR (-0.1%) and fasting glucose-to-insulin ratio compared with placebo group (0.0%) (28).

*Orlistate:* A more recent study revealed a significant effect for combination of orlistat with lifestyle modification on weight and HOMA-IR in both obese PCOS and non-PCOS subjects (45).

Due to the scarcity of data on the combination of thiazolidinediones with lifestyle modification, this review was unable to generate a recommendation while insisting on possible benefits for this combination compared with the results of single treatment with rosiglitazone. Furthermore due to the lack of studies that assessed the effect of treatments with OCP on normal weight subjects and combination of metformin and thiazolidendios on overweight subjects, this review was not able to compare the effect of these treatment modalities on subjects with normal weight and overweight or obese subjects.

## Discussion

This study revealed that each treatment option in PCOS has a specific effect on the prevention of diabetes. The preventive effect of diet is due to its main nutrient constituents including carbohydrates, fats, fiber and protein (46). It was shown that inclusion of low GI carbohydrates and high fiber diets as well as high fat low carbohydrate diet can result in reduced risk of diabetes (46). Knowing that poly-unsaturated fatty acids (PUFA) improves insulin resistance; therefore type of fat other than the type of dietary carbohydrate can be a determinant of the anti-diabetic properties of a specific diet (46). On the other hand, physical activity was shown to reduce BMI as well as improve emotional status in over weight and obese women (47, 48). Regarding high dropout rate in maintaining a low calorie diet, it seems logical to combine diet with exercise in order to improve the effect of lifestyle intervention (11, 16). 

Pharmacological treatments were also shown to have preventive effects on DM in PCOS. Metformin is a widely used anti-diabetic drug and has been administered frequently in PCOS subjects (15, 18-21, 39). This review indicated that metformin is the only pharmacological treatment option that has the capability of reducing WHR, BMI, FBS as well as increasing insulin sensitivity(28, 29, 49). Furthermore, metformin has higher efficacy in reducing the risk of diabetes at higher doses (15, 18, 20, 21, 39). Besides, it was shown that overweight or obese PCOS subjects may present different responses to metformin administration compared with normal weight PCOS subjects (41). 

This review found that higher doses of metformin along with lifestyle modification may result in increased insulin sensitivity and prevent diabetes in obese PCOS subjects while more studies should be performed on non-obese PCOS subjects in order to observe whether combination therapy can improve the protective effects of metformin against diabetes. Moreover, based on the positive effects of lifestyle modification on other causes of morbidity and mortality it is recommended that lifestyle modification should be included in treatment protocol of PCOS subjects (4, 11, 12, 14).

This review indicated that although TDZs can result in increased insulin sensitivity, they have no effect on weight and WHR (fat distribution). Although there is a scarcity of publication on the effects of TDZs in the prevention of diabetes, TZDs are also considered as treatment options in PCOS especially when weight reduction is not achievable (4). More studies should be performed to assess the effects of TDZs in contrast with metformin and other treatment option amongst non-obese and obese PCOS subjects. 

Only one study compared the effect of combination treatment with metformin and TDZ in terms of diabetes risk reduction in PCOS (28). The findings of the study revealed that although combination of metformin and TZD at their therapeutic doses did not result in weight loss but resulted in fat mobilization and reduced WHR, which was not observed in the mono-therapy (28). More studies should be conducted to identify whether combination therapy with metformin and TDZ can result in the administration of these drugs at lower doses.

Orlistat is amongst other treatment options available for PCOS subjects. This study found that the effect of orlistat in weight reduction might be similar to metformin but the meta-analysis that reported this finding was based on the assessment of 2 studies, therefore; more studies are needed to identify the benefits of orlistat (27, 34, 38).

The findings of this review strengthen the findings of a previous review. In the review by Sharma et al. (2006) three recommendations were suggested for prevention of DM in PCOS subjects. Firstly the PCOS should be diagnosed in early stages of infertility, secondly all PCOS patents above the age of 30 years should undergo DM screening with 75 g oral glucose, since fasting blood sugar might not be altered prior to establishment of DM, and thirdly lifestyle modification and insulin sensitisers should be prescribed for PCOS subjects (6). 

One of the strengths of this review was the comparison of the response to treatment in non-obese PCOS subjects with overweight and obese PCOS subjects. Moreover, this review found a possible synergistic effect for the combination of lifestyle modification with medical treatments that might result in better compliance and improved outcomes.

***Conclusion:*** This systematic review revealed that lifestyle interventions are effective in reducing BMI in PCOS women but due to the difficulty in maintaining low calorie diet, lifestyle interventions should be accompanied with exercise. Furthermore, metformin was found to be effective in improving the indicators of DM in PCOS women especially if accompanied with lifestyle modification. 

Due to the scarcity of data on these treatment modalities, no recommendation could be generated for prescription of the assessed medications to reduce the risk of DM in women with PCOS.
